# Comment on “Evaluation of Antiviral Therapy Performed after Curative Therapy in Patients with HBV-Related Hepatocellular Carcinoma: An Updated Meta-Analysis”

**DOI:** 10.1155/2016/7625982

**Published:** 2016-07-14

**Authors:** Han-Yue Mo, Bang-De Xiang, Jian-Hong Zhong, Le-Qun Li, Xue-Mei You

**Affiliations:** Hepatobiliary Surgery Department, Affiliated Tumor Hospital, Guangxi Medical University, Nanning 530021, China

We read with interest the updated meta-analysis by Yuan et al. [1] which investigated the role of nucleos(t)ide analog (NA) therapy for patients with hepatitis B virus (HBV) related hepatocellular carcinoma (HCC) after surgery. Even after curative resection or ablation, the incidence of HCC recurrence is very high [2]. However, no adjuvant and/or postoperative therapies with definite efficacy to prevent HCC recurrence are recommended by official guidelines. HBV is the main cause of HCC in Asia. In this point, Yuan and coworkers [1] performed an interesting and important updated meta-analysis in order to determine whether antiviral therapy with NA can prevent HCC recurrence and improve patients' survival after surgery. Their meta-analysis showed that NA therapy significantly reduced 1- and 3-year recurrence and significantly improved 1-, 3-, and 5-year overall survival and disease-free survival. We feel that some of these results may need to be treated with caution. And we would like to propose several important issues raised in this meta-analysis.

The first drawback of this meta-analysis is using a controversial index, which is relative risk, which is used to reflect the cumulative risk of the whole experiment. However, tumor recurrence and mortality may occur at any time during the whole experiment. Therefore, the best index in survival analyses is hazard ratio, which reflects instantaneous risk at each point of survival curve. Second, intention-to-treat analysis was not used. The same total number of patients in each study was used to calculate tumor recurrence and overall and disease-free survival analyses at 1, 3, and 5 years. Actually, most included patients are destined to experience tumor recurrence or die during follow-up. Third, the definition of disease-free and recurrence-free survival was confusing. And fourth, it is better to perform subgroup analysis only based on randomized trials [3, 4].

Though the exact mechanism of how NA therapy prevents HCC recurrence and improves overall survival is unknown, it is said that NA therapy has no direct antitumor effect. It may inhibit hepatitis activity and reduce chronic inflammation in the remnant liver to improve hepatic functional reserve after surgery and then increase the chances for further treatment. Moreover, prolonged suppression of HBV replication may reduce the risk of HBV-related HCC development. The effect of NA therapy will be observed in a long-term follow-up. Theoretically, postoperative NA therapy has no impact on patients' short-term survival. Therefore, the results that the 1-year tumor recurrence and overall and disease-free survivals were significantly better in the NA group may not be true. More obviously, difference in the 3 and 5 years may be the result of the long-term effect of NA therapy.

Even though the present meta-analysis revealed that NA can reduce the recurrence and improve the prognosis of HBV-related HCC after surgery, the following issues still need to be addressed in further studies [5]:

- What are the indications for NA therapy?
- Which NA drug(s) should be used?
- When is the optimal time to initiate NA therapy, and how long should it last?
- Can multimodal treatment improve on NA therapy?

Only when these issues are answered, the role of antiviral therapy with NA after surgery would be definite.
